# A new species and new record of the cryptobiotic ant genus *Ponera* Latreille, 1804 (Hymenoptera, Formicidae) from Hong Kong

**DOI:** 10.3897/zookeys.867.36139

**Published:** 2019-07-29

**Authors:** Mac P. Pierce, Chi-Man Leong, Benoit Guénard

**Affiliations:** 1 The University of Hong Kong, School of Biological Sciences, Kadoorie Biological Sciences Building, Pok Fu Lam Road, Hong Kong SAR, China The University of Hong Kong Hong Kong China

**Keywords:** Asia, biodiversity, description, Ponerinae, taxonomy

## Abstract

Despite its small size, Hong Kong hosts a surprising level of ant diversity. Through faunal studies on arthropods conducted in Hong Kong over recent years, a new record and species of the genus *Ponera* have been discovered, which are introduced here. *Ponera guangxiensis* Zhou, 2001 is reported for the first time from Hong Kong, and *Ponera tudigong***sp. nov.** is here described as a new species, easily distinguishable from other *Ponera* species and unique within the genus for its four mandibular teeth.

## Introduction

The cryptic ant genus *Ponera* Latreille, 1804, contains 59 valid species at present (Bolton 2019; Leong et al. 2019). Known mostly from the Old World, the bulk of its diversity occurs in the tropical to subtropical Oriental realm (Janicki et al. 2016; Guénard et al. 2017). The tropical diversity of *Ponera* in the Oriental realm and further south into the Oceanian and Australian realms contrasts with its absence as native species in the Afrotropical and Madagascan realms, and its limited distribution within the Neotropical realm, where it is limited to two species in the northern part of Mesoamerica (Branstetter and Longino 2019). Relatively little is known of the biology and ecology of the species of *Ponera*, owing to its cryptobiotic lifestyle and paucity of collection. In general, *Ponera* has small colonies (ca. hundreds of individuals), is most commonly found underneath rocks and rotting logs, and is likely a generalist predator of small arthropods (Schmidt and Shattuck 2014).

At present only one species of *Ponera*, *P. sinensis* Wheeler, 1928, is known from Hong Kong and the surrounding Guangdong province. Through faunal studies conducted in Hong Kong using pitfall traps and sifted leaf-litter, two newly recorded species have been found: *P. guangxiensis* Zhou, 2001, and *P. tudigong* sp. nov., newly described in this paper. We include here accounts of the two species, the description of *P. tudigong* sp. nov., and an update to the key to East Asian species of *Ponera* presented by Leong et al. (2019).

## Materials and methods

### Material examined

**Figure 1.**
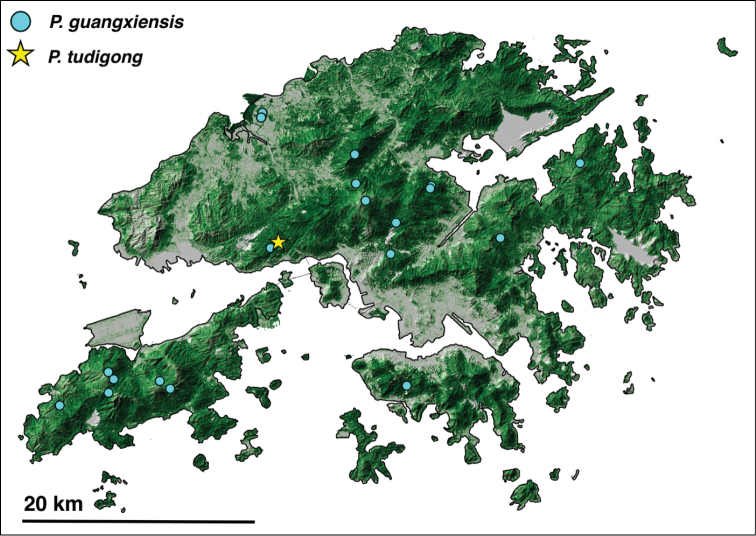
Topographic map of Hong Kong, with tree cover shown in green (darker color indicates greater tree cover, lighter color indicates less). Collection records for Hong Kong *Ponera* species are: *P. guangxiensis* (light-blue circle), *P. tudigong* sp. nov. (yellow star).

Twenty-nine individuals collected from Hong Kong were examined (Figure 1). All non-type material has been deposited in the Insect Biodiversity and Biogeography Laboratory (**IBBL**) at the University of Hong Kong. The type specimens of *P. tudigong* have been deposited at the California Academy of Sciences (**CAS**). Images and specimen data were uploaded to AntWeb.org (2018) and are available in the supplementary specimen file.

### Morphology

Morphological measurements were done using a Leica S8AP0 stereomicroscope at 80 × magnification. Measurements used are detailed below and follow those used in Leong et al. (2019), with the exception of the addition of mandible length (MaL) and change of total length (TL) which are described below. Weber’s length (WL) has been changed to mesosomal length (ML). Measurements are reported in millimeters to two decimal places. Repeated measurements on a single individual resulted in an accuracy of 0.01 mm. Individuals of *Ponera guangxiensis* from different regions of Hong Kong were measured to account for population variability. Terminology follows Taylor (1967), Harris (1979), Bolton (1994), and Keller (2011).

### Imaging

Specimen images were taken using a Leica DFC450 digital camera mounted on a Leica M205C dissecting microscope, and processed using Leica Application Suite V4 software.

**ATL** Abdominal tergum III length. Maximum length of 3^rd^ abdominal tergum (= 1^st^ gastral tergum), measured from the center of the anterior margin to the center of the posterior margin, in dorsal view.

**ATW** Abdominal tergum III width. Maximum width of the 3^rd^ abdominal tergum (= 1^st^ gastral tergum), measured as a straight line from one lateral margin to the other, in dorsal view.

**HL** Head length. Maximum length of the head, measured as a straight line between the center of the posterior cephalic margin and the center of the anterior clypeal margin, in full-face view.

**HW** Head width. Maximum width of the head, measured as a straight line from one lateral margin to the other, in full-face view.

**MaL** Mandible length. Maximum length of the mandible, measured as a straight line from the mandibular insertion to the tip of the apical tooth, with the mandible in dorsal view.

**ML** Mesosomal length. Maximum length of the mesosoma, measured as a straight line from the anterior-most point of the pronotum to the posterior basal angle of the metapleuron, in profile view.

**PeH** Petiole height. Maximum height of the petiole, measured as a straight line from the ventral margin of the subpetiolar process to the dorsal margin of the petiole, in profile view.

**PeNL** Petiole node length. Length of the node of the petiole, measured as a straight line from the anterior margin of the petiole immediately above the dorsal base of the anterior petiolar tubercle to the posterior margin of the petiole, in profile view.

**PeW** Petiole width. Maximum width of the petiole, measured as a straight line from one lateral margin of the petiole to the other, in dorsal view.

**PrW** Pronotal width. Maximum width of the pronotum, measured as a straight line from one lateral margin of the pronotum to the other, in dorsal view.

**SL** Scape length. Maximum length of the scape, measured as a straight line from the base of the scape (excluding the basal neck and condyle).

**TL** Total length. Maximum horizontal length of the specimen from the anterior-most extent of the mandibles to the posterior tip of the gaster, measured in profile view.

**ATI** Abdominal tergum III (= 1^st^ gastral tergum) index. ATL/ATW × 100.

**CI** Cephalic index. HW/HL × 100.

**DPeI** Dorsal petiole index. PeW/PeNL × 100.

**LPeI** Lateral petiole index. PeNL/PeH × 100.

**PeI** Petiole index. PeW/PrW ×100.

**SI** Scape index. SL/HW × 100.

## Species accounts

### Ponera tudigong sp. nov.

Taxon classificationAnimaliaHymenopteraFormicidae

e3d0c2d3-fb56-5470-8291-58947b6dee4d

http://zoobank.org/37401E74-D228-4D0B-AD52-995EDF69C40A

**Figure 2.**
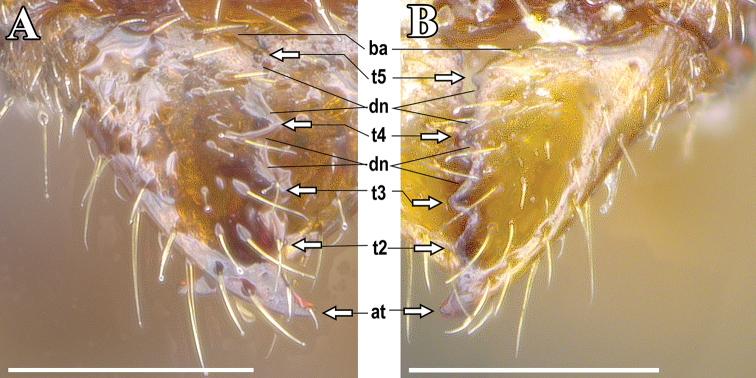
Mandible of *Ponera tudigong* sp. nov. **A** Queen (ANTWEB1009670) **B** worker (ANTWEB1009796). Abbreviations: ba: basal angle of mandible; t: tooth number; at: apical tooth of mandible; dn: denticle; sensu Bolton (1994).

**Figure 3.**
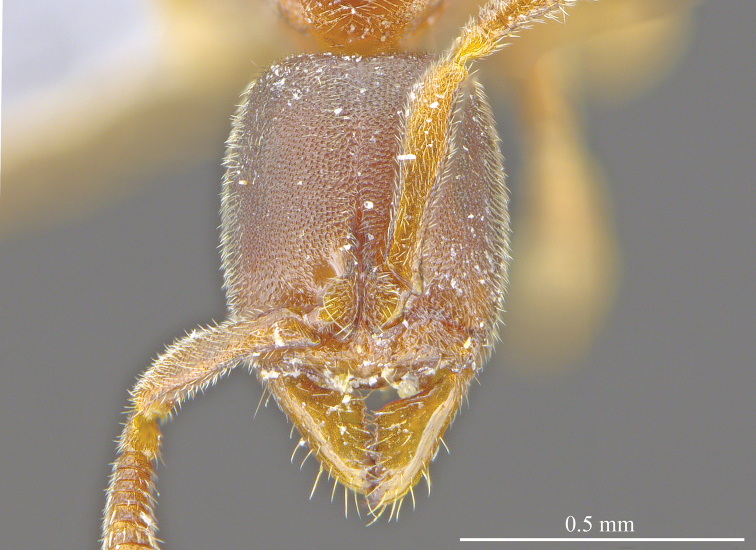
*Ponera tudigong* sp. nov., worker (ANTWEB1009796). Head in full-face view.

**Figure 4.**
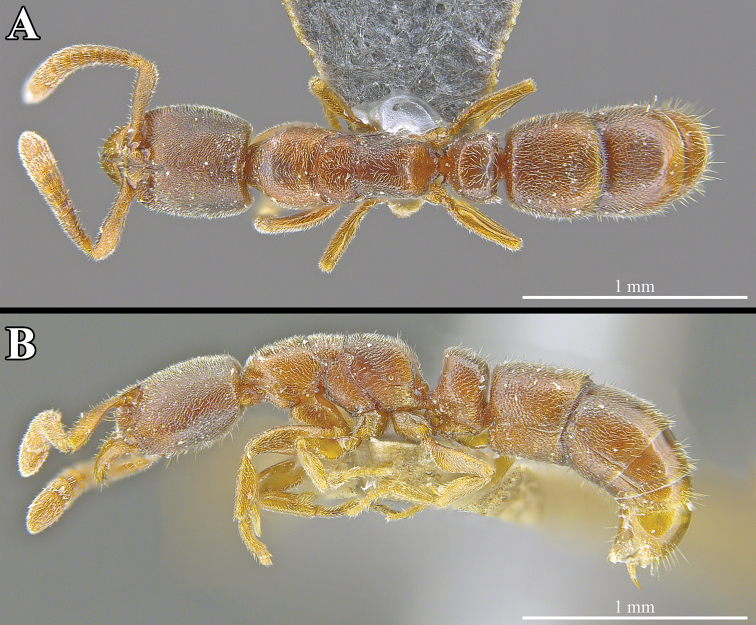
*Ponera tudigong* sp. nov., worker (ANTWEB1009796). **A** Dorsal view **B** lateral view.

**Figure 5.**
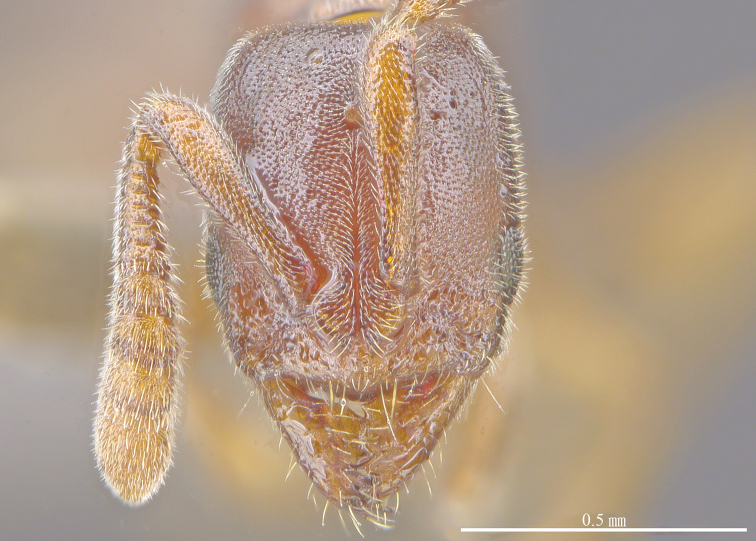
*Ponera tudigong* sp. nov., queen (ANTWEB1009670). Head in full-face view.

**Figure 6.**
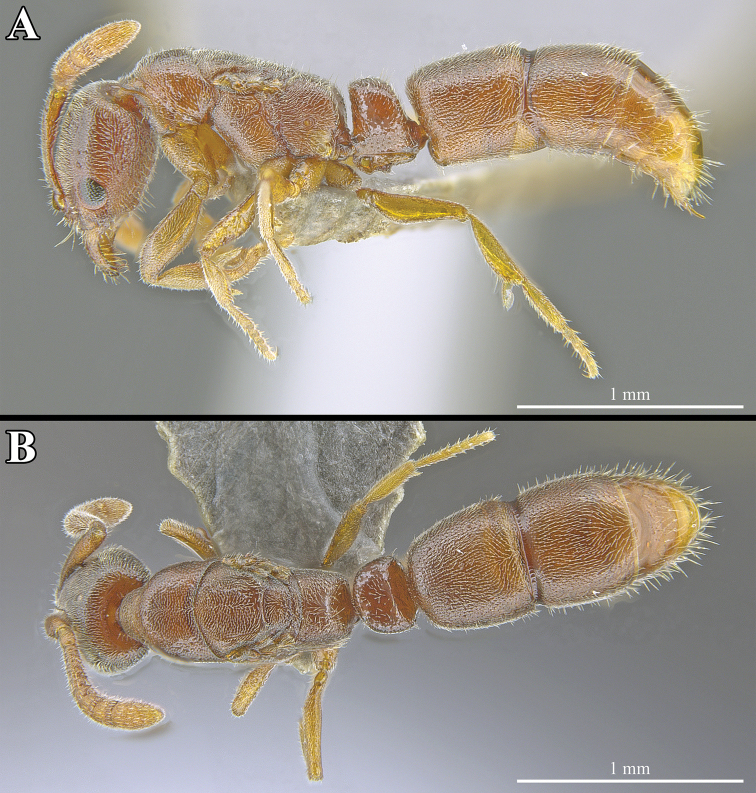
*Ponera tudigong* sp. nov., queen (ANTWEB1009670). **A** Lateral view **B** dorsal view.

Figs 2 , 3 , 4 , 5 , 6

#### Type material.

Holotype, worker: Hong Kong Special Administrative Region, Tai Lam Country Park, 22.38079N, 114.05378E, ± 45 m, 249 m above sea level (a.s.l.), 8 November 2017 (collection code Pi-MPP-43-07, pitfall) [CAS, unique specimen identifier CASENT0872070].

#### Non-type material.

Queen: same data as holotype worker (collection code Pi-MPP-43-05, pitfall) [CAS, CASENT0872071].

#### Geographic range.

Hong Kong S.A.R., China.

#### Diagnosis (worker).

*Ponera tudigong* can be distinguished from the other species in the genus by the following characters: masticatory margin of the mandible with a small denticle and four enlarged teeth (Figure 2), an apomorphic character for this new species (see Leong et al. 2019); petiole relatively thick in lateral view (LPeI 60) with the anterodorsal margin of the node of the petiole projecting slightly forward; metanotal groove forming clearly incised suture in dorsal view (Fig. 4A); eyes absent.

#### Measurements and indices (mm).

Worker (n = 1): ATL 0.43, ATW 0.46, HL 0.59, HW 0.51, MaL 0.29, ML 0.82, PeH 0.39, PeNL 0.23, PeW 0.32, PrW 0.38, SL 0.43, TL 2.3. Indices: ATI 92, CI 85, DPeI 138, LPeI 60, PeI 85, SI 84.

Queen (n = 1): ATL 0.54, ATW 0.54, HL 0.65, HW 0.56, MaL 0.41, ML 1.03, PeH 0.43, PeNL 0.25, PeW 0.35, PrW 0.47, SL 0.48, TL 2.5. Indices: ATI 101, CI 85, DPeI 140, LPeI 59, PeI 75, SI 85.

#### Description of worker.

*Head.* In full face view, head subrectangular (Fig. 3), longer than broad (CI: 85) with shallowly concave occipital margin, and slightly convex lateral margins. Anterior clypeal margin nearly straight, with a low and blunt medial tooth. Eyes absent. Mandibles subtriangular, with a small denticle (*t5*) and four triangular enlarged teeth (*t1* to *t4*) on the masticatory margin (Fig. 2A). Antennal scapes, when laid back, nearly reaching occipital corners of head; average ratio of the length of antennomeres

Funicular segments of antennae incrassate, increasing in length and breadth distally.

*Mesosoma.* In profile view, mesosomal dorsum slightly convex. Promesonotal suture and metanotal groove both present and clearly incised. Mesopleuron distinct, separated from mesonotum by a clearly incised promesonotal articulation. In dorsal view, lateral margins of pronotum and mesonotum rounded, lateral margins of propodeum slightly concave. Propodeal corner in lateral view forming a blunt angle.

*Metasoma.* In profile view, petiole sub-rectangular and remarkably thick compared to other species in the genus (LPeI = 59.5), with anterior and posterior margins straight and parallel; anterodorsal corner nearly right-angled, posterodorsal corner blunt; anterodorsal corner higher than posterodorsal corner. Subpetiolar process sub-rectangular in shape, with a large and circular anterior fenestra, extending across the entire ventral margin of the petiole. Posterior portion of subpetiolar process with a well-developed pair of blunted teeth. In dorsal view, petiolar node sub-oval, clearly wider than long (DPeI = 138.1), with a slightly convex anterior margin and slightly concave posterior margin. In dorsal view, abdominal tergum III (= 1^st^ gastral tergum) wider than long (ATI = 91.8), and in profile view with a bluntly rounded anterodorsal corner.

*Sculpture.* Entirety of head punctate, covered by closely and evenly spaced fine puncturing. Mandibles smooth. Sculpturing of mesosoma and metasoma relatively light, ranging from puncticulate to imbricate. Mesosomal and metasomal dorsum nearly smooth.

*Pilosity.* Head and antennae covered by fine golden-colored pubescence. Mandibles with sparse, short, erect to sub-erect filiform hairs. Pilosity of mesosoma consisting of a fine pubescence, similar to that of the head. Fine and sparse pubescence covering each metasomal segment, becoming sparser after the fourth abdominal segment (= 2^nd^ gastral segment). Short, golden, sub-erect to erect filiform hairs present on all metasomal segments, becoming longer and denser posteriorly from the fifth abdominal segment (= 3^rd^ gastral segment).

*Color.* Coloration of entire body of the individual, excluding the legs, ranging from light to dark reddish-brown. Legs light brown.

#### Description of queen.

The description of the queen of *P. tudigong* follows that of the worker caste, with the following differences:

Body size larger than that of the worker caste, with large, sub-circular compound eyes, 0.15 mm in diameter, present on the anterior part of the head, roughly one-third the distance to the posterior margin. Three distinct ocelli present on the posterior part of the head. Mandibles similar to worker caste (Fig. 2).

Sub-petiolar process of the queen with a subquadrate anterior fenestra, a low and blunt downward-projecting anterior angle, and a downward-projecting square medial tooth. Abdominal tergum III (= 1^st^ gastral tergum) as long as wide (ATI: 101).

Sculpturing, coloration, and pilosity of the queen is similar to that of the worker caste, with sculpturing slightly more pronounced.

#### Etymology.

This new species is named in honor of Tudigong (土地公), the lord of the soil and the ground, a widely venerated Chinese deity.

#### Comments.

*Ponera tudigong* is known from a single location in Tai Lam country park, Hong Kong SAR. The worker and queen were collected in separate pitfall traps 5 meters apart during the same sampling period. They were initially recognized as being distinct from the other local *Ponera* species, *P. sinensis* and *P. guangxiensis*, based on the mandible and shape of its petiole and the absence of eyes, and was later determined to be a new species. It is the only known *Ponera* species with four mandibular teeth, all other species having either three (most of *Ponera* species), five (*P. pentodontos* Xu, 2001), or seven (*P. taylori* Bharti & Wachkoo, 2012).

Nothing is known about the biology or life history of *P. tudigong*. Like most species of *Ponera*, it is likely cryptobiotic and subterranean, as the absence of eyes might suggest. The fact that our specimens were captured in surface pitfall traps may suggest that the species does come to the surface at least occasionally, maybe at night, and possibly to forage or for reproduction purposes, given that a wingless queen was captured in addition to the worker. Of course, soil disturbance during trap emplacement may also be responsible.

*Ponera tudigong* is known from only a single collection event, despite a significant amount of leaf-litter and pitfall-trap sampling done throughout Hong Kong in recent years. This suggests that the species is indeed quite rare. The habitat it was collected from is a restored secondary forest, and it was collected within one meter of a well-used hiking trail.

### Ponera guangxiensis

Taxon classificationAnimaliaHymenopteraFormicidae

Zhou, 2001

bce8e7c5-af9d-5978-b76f-6f1ec96422ff

Fig. 7

Ponera guangxiensis Zhou, 2001A: 37, 227, figs 33, 34 (w.) CHINA. Indomalaya.

#### Geographic range.

China: Guangxi province, Hong Kong S.A.R (new record); Vietnam.

#### Measurements and indices (mm).

Worker (n = 6): ATL 0.38–0.44, ATW 0.48–0.51, HL 0.54–0.58, HW 0.49–0.53, MaL 0.31–0.36, ML 0.74–0.81, PeH 0.38–0.41, PeNL 0.19–0.22, PeW 0.31–0.32, PrW 0.38–0.40, SL 0.38–0.42, TL 2.19–2.38. Indices: ATI 79–91, CI 85–94, DPeI 143–167, LPeI 46–54, PeI 78–84, SI 75–86.

Queen (n = 1): ATL 0.50, ATW 0.57, HL 0.59, HW 0.53, MaL 0.34, ML 0.91, PeH 0.44, PeNL 0.21, PeW 0.38, PrW 0.46, SL 0.44, TL 2.55. Indices: ATI 88, CI 89, DPeI 182, LPeI 46, PeI 82, SI 85.

#### Diagnosis.

See Leong et al. (2018).

**Figure 7.**
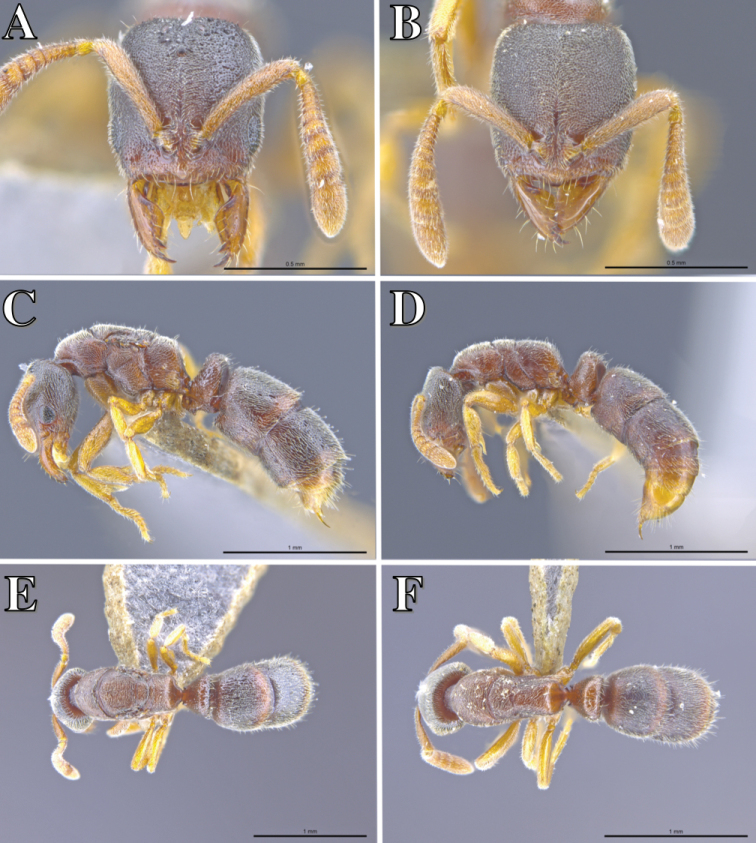
*Ponera guangxiensis* Zhou, 2001, worker (ANTWEB1016982) and queen (ANTWEB1016609). **A** Queen head, full-face view **B** worker head, full-face view **C** queen, lateral view **D** worker, lateral view **E** queen, dorsal view **F** worker, dorsal view.

#### Material examined.

Hong Kong Special Administrative Region (HKSAR), Kam Shan Country Park, New Territories, 22.37089N, 114.14839E, 18 October 2017 (KS S1-R, Winkler sifter) [IBBL, ANTWEB1016325]. HKSAR, Tai Lam Country Park, New Territories, 22.37598N, 114.04713E, 3 November 2017 (TL S2-C (B), Winkler sifter) [IBBL, ANTWEB1016405]. HKSAR, Ngong Ping, Lantau Island, 22.25403N, 113.9105E, 9 November 2017 (NP S1-C, Winkler sifter) [IBBL, ANTWEB1016486]. HKSAR, Ngong Ping, Lantau Island, 22.26537N, 113.91491E, 9 November 2017 (NP S2-C, Winkler sifter) [IBBL, ANTWEB1016506]. HKSAR, Ngong Ping, Lantau Island, 22.27144N, 113.9103E, 9 November 2017 (NP S3-C, Winkler sifter) [IBBL, ANTWEB1016519] (1 worker, 1 dealate queen). HKSAR, Pak Sha O, New Territories, 22.44743N, 114.3082E, 17 November 2017 (PSO S1-R, Winkler sifter) [IBBL, ANTWEB1016555] (1 worker, 1 dealate queen). HKSAR, Ling Wui Shan, Lantau Island, 22.24333N, 113.86932E, 4 December 2017 (LWS S1-C, Winkler sifter) [IBBL, ANTWEB1016609]. HKSAR, Ling Wui Shan, Lantau Island, 22.24333N, 113.86932E, 4 December 2017 (LWS S1-R, Winkler sifter) [IBBL]. HKSAR, Ngong Ping, Lantau Island, 22.25403N, 113.9105E, 9 November 2017 (NP S1-R, Winkler sifter) [IBBL]. HKSAR, Ngong Ping, Lantau Island, 22.25403N, 113.9105E, 9 November 2017 (NP S3-R, Winkler sifter) [IBBL] (1 worker, 1 dealate queen). HKSAR, Tai Lam Country Park, New Territories, 22.37598N, 114.04713E, 3 November 2017 (TL S2-R, Winkler sifter) [IBBL]. HKSAR, Ngong Ping, Lantau Island, 22.25396499N, 113.910478E, 428 m a.s.l., 9 November 2017 (IAS-0162, hand collected) [IBBL, ANTWEB1009802; ANTWEB1009629; ANTWEB1009803] (3 workers). HKSAR, Tai Po Kau, New Territories, 22.42833N, 114.18273E, 171 m a.s.l., 2 June 2017 (IAS-0215, hand collected) [IBBL, ANTWEB1016982; ANTWEB1009685; ANTWEB1016939] (3 workers). HKSAR, Tai Po Kau, New Territories, 22.426138N, 114.181783E, 14 July 2015 (RHL-HK-TPK-T1WM, Winkler sifter) [IBBL, RHL00642]. HKSAR, Sunset Peak, Lantau Island, 22.263923N, 113.953762E, 3 June 2015 (RHL-HK-LSP-T3WM, Winkler sifter) [IBBL, RHL00758]. HKSAR, Tai To Yan, New Territories, 22.454795N, 114.118215E, 7 August 2015 (RHL-HK-TYF-T1WJ, Winkler sifter) [IBBL, RHL01441]. HKSAR, Tai To Yan, New Territories, 22.454795N, 114.118215E, 7 August 2015 (RHL-HK-TYF-T1WM, Winkler sifter) [IBBL, RHL01467]. HKSAR, Aberdeen Reservoir, Hong Kong Island, 22.26N, 114.162E, 26 June 2015 (RHL-HK-ABR-T4WM, Winkler sifter) [IBBL, RHL02059]. HKSAR, Kadoorie Farm and Botanical Garden, New Territories, 22.4302N, 114.1192E, 14 September 2015 (RHL-HK-KFBG-T1WM, Winkler sifter) [IBBL, RHL02550]. HKSAR, Mau Ping Wood, New Territories, 22.3844N, 114.241E, 20 October 2015 (RHL-HK-MTLW-T1W J, Winkler sifter) [IBBL, RHL02599]. HKSAR, Mai Po Nature Reserve, New Territories, 22.49N, 114.04E, 10 m a.s.l., 13 September 1993 [KFBG, ANTWEB1015483; ANTWEB1015484] (2 workers). HKSAR, Sunset Peak South, Lantau Island, 22.2573N, 113.9622E, 670 m a.s.l., 8 October 1996 (Winkler sifter) [KFBG, ANTWEB1016030]. HKSAR, Mai Po Nature Reserve, New Territories, 22.486N, 114.039E, 10 m a.s.l., 13 September 1993 (Pitfall and bait). HKSAR, Shing Mun Reservoir, New Territories, 22.39718N, 114.15273E, 230 m a.s.l., 6 July 2011 (PSW16625-05, Winkler sifter). HKSAR, Tai Mo Shan, New Territories, 22.41595N, 114.12722E, 775 m a.s.l., 5 July 2011 (PSW16616-03, Winkler sifter).

#### Comments.

*Ponera guangxiensis* is relatively common in collections within Hong Kong. It is most commonly collected in native secondary forests, either in leaf litter samples or by searching under stones or rotting logs. It appears to be widespread in Hong Kong, though no observations on its biology or ecology have been made.

### Ponera sinensis

Taxon classificationAnimaliaHymenopteraFormicidae

Wheeler, 1928

ce3f980d-ec93-5c6a-9766-594ed651900b

Ponera sinensis Wheeler, 1928c: 6 (w.) CHINA (Hong Kong). Indomalaya.

#### Geographic range.

Guangxi province, Yunnan province, Taiwan, Hong Kong S.A.R.

#### Diagnosis.

See Wheeler (1928), Wilson (1957), Taylor (1967), and Leong et al. (2019).

#### Comments.

To the best of our knowledge, the only confirmed specimen of *P. sinensis* from Hong Kong is the holotype, collected by Professor F. Silvestri from an unknown location, and described by Wheeler (1928). Despite numerous faunal studies in Hong Kong over recent years, no further specimens of *P. sinensis* have been identified. On the contrary, the vast majority of *Ponera* specimens collected from Hong Kong represent the newly recorded *P. guangxiensis*. Whether this represents an underlying ecological change or simply an artefact of sampling remains unclear, and future sampling in Hong Kong may yield new specimens of *P. sinensis*.

### Update to the identification key of East Asian *Ponera* species

The following is an update to the identification key to East Asian species of *Ponera* originally provided by Leong et al. (2019), which contains 31 key couplets for 32 species. *Ponera tudigong* sp. nov., described here, is placed in a new couplet inserted after the 4^th^ couplet in Leong et al. (2019), which has been slightly modified to reflect this. The new couplet has been numbered as 4’, to discern it from couplet 4 in the original key, and to leave the remainder of the original key unaffected.

**Table:** 

1	In lateral view, petiolar node very thick with convex posterior margin; with upper portion of posterior margin bulging	**2**
–	In lateral view, petiolar node not as developed and presenting straight to convex posterior margin; with upper portion of posterior margin not bulging	**3**
2	Petiolar node in dorsal view with slightly convex anterior margin and slightly concave posterior margin. Smaller species, HW: 0.54–0.60 mm. Body color dark	***P. rishen* Terayama, 2009**
–	Petiolar node in dorsal view with strongly convex anterior margin and strongly concave posterior margin. Larger species, HW: 0.65–0.75 mm. Body color reddish brown	***P. takaminei* Terayama, 1996**
3	Eye large, each consisting of 20 or more facets. Larger species, HW: ca. 0.68 mm	***P. kohmoku* Terayama, 1996**
–	Eye small, each consisting of 10 or fewer facets. Size variable	**4**
4	Masticatory margin of mandible with 5 subequal large teeth. Smaller species, HW: 0.53–0.55 mm	***P. pentodontos* Xu, 2001**
–	Masticatory margin of mandible with 3 or 4 enlarged teeth. Size variable	**4**’
4’	Masticatory margin of mandible with 4 enlarged teeth followed by a small denticle	***P. tudigong* sp. nov.**
–	Masticatory margin of mandible with 3 enlarged teeth followed by a series of small to indistinct denticles; the number of denticles variable	**5**

## Supplementary Material

XML Treatment for Ponera tudigong

XML Treatment for Ponera guangxiensis

XML Treatment for Ponera sinensis
